# The psychosocial health of sexual and gender minority people with anal and colorectal cancer: a mixed methods study

**DOI:** 10.1007/s11764-024-01611-5

**Published:** 2024-05-08

**Authors:** Oscar Y. Franco-Rocha, Katie Trainum, Christopher W. Wheldon

**Affiliations:** 1 The University of Texas at Austin, Austin, TX USA; 2 Temple University, Philadelphia, PA USA

**Keywords:** Anal cancer, Colorectal cancer, Oncology, Sexual and gender minorities, Psycho-oncology, Healthcare disparities

## Abstract

**Purpose:**

Sexual and gender minority (SGM) cancer survivors have poorer psychosocial health than their heterosexual cisgender counterparts. Nevertheless, most research has focused on breast and prostate survivors. It is unknown how different gastrointestinal (GI) cancers affect the psychosocial well-being of SGM individuals. We (1) described the psychosocial health of SGM people with GI cancers and (2) identified differences in psychosocial health outcomes by cancer type.

**Methods:**

We conducted a concurrent mixed-methods secondary analysis with identical samples (*n* = 295) using data from “OUT: The National Cancer Survey.” Likert-type and open-ended questions about demographics, satisfaction with care, social support, and access to mental health resources were included in the analysis. Poor mental health was the primary outcome. Quantitative (linear regression) and qualitative (thematic analysis) data were independently analyzed, then integrated through the narrative, weaving approach.

**Results:**

Three concepts emerged after data integration, (1) positive and negative influences on the psychological well-being of the participants; (2) social adaptations with the participants’ friends, partners, and family members; and (3) additional factors affecting the psychosocial well-being during and after cancer, particularly interactions with providers, comorbidities, and treatment side effects.

**Conclusion:**

Clinical characteristics, demographic factors, and culturally responsive care influenced the psychosocial health of SGM survivors of GI cancers.

**Implications for Cancer Survivors:**

Participants developed their own coping strategies and advocated for the SGM community. Interventions promoting peer support and self-esteem are a priority for this population. Healthcare professional training should incorporate historical trauma awareness and assess the delivery of culturally responsive care.

**Supplementary Information:**

The online version contains supplementary material available at 10.1007/s11764-024-01611-5.

## Introduction

Although epidemiologic data to analyze disparities in gastrointestinal (GI) cancer incidence, prevalence, and mortality among sexual and gender minority (SGM) individuals is limited [1], it is well known that risk factors for GI cancers, such as colorectal and anal, disproportionately affect SGM populations [2]. Sexual minority men (SMM) are more likely to be diagnosed with human immunodeficiency virus (HIV) and human papillomavirus (HPV) and have higher rates of substance use than their heterosexual cisgender counterparts. Similarly, sexual minority women (SMW) tend to exercise less and have higher rates of obesity than heterosexual cisgender women. All of which are risk factors for developing GI cancers [3–7].

During and after cancer treatment, SGM individuals tend to experience worse psychosocial outcomes (e.g., anxiety, poorer social support) than heterosexual cisgender cancer survivors due to structural factors that privilege cisgender and heterosexual identities [8–11]. Even though studies have extensively analyzed the psychosocial health of SGM people with cancer, most focus on people diagnosed with breast and prostate cancer [10, 12]. Despite the high prevalence of GI cancers, studies analyzing its impact on the psychosocial health of SGM individuals are scarce.

Boehmer et al. [13, 14] found that sexual minority people with colorectal cancer were more likely to experience anxiety and worse mental health than their heterosexual cisgender peers. Although these differences were no longer significant after adjusting for social, contextual, and psychological factors, the authors hypothesize that individuals’ use of mental healthcare services before cancer diagnosis and previous experiences of discrimination may have attenuated the contribution of sexual orientation to mental health in the statistical models [13]. Furthermore, younger age, anxiety, depression, and embarrassment due to having a stoma were unique contributors to the mental health of sexual minority people with colorectal cancer [15]. Through qualitative inquiry, this population also expressed challenges with social support, economic stability, and interacting with healthcare providers [16]. Finally, in a study conducted in Brazil with 19 SMM with anal cancer, Mauro et al. [17] found that this population experiences worse quality of life and sexual functioning during and after cancer treatment compared to pre-treatment scores.

Although the previous studies shed light on the psychosocial health of SGM people with some GI cancers, they were limited to cisgender participants [13–15] with small SGM sample sizes [17]. The present study expands on this body of literature by including sexual minority people and transgender and non-binary (TGNB) individuals from a nationwide survey study conducted in the USA. In this study, we aimed to (1) describe the psychosocial health of SGM people with GI cancers and (2) identify differences in psychosocial health outcomes among SGM people with colorectal or anal cancers.

We conducted a concurrent mixed-methods secondary analysis with identical samples [18]. The purpose of using a mixed-methods design is to converge quantitative and qualitative data to gain a holistic understanding of cancer’s impact on the psychosocial health of SGM individuals with GI cancers [19]. Combining both types of data helped us capture, expand, and contrast different aspects of the phenomena of study [20]. Quantitative and qualitative data were assigned the same weight throughout data analysis and interpretation. The questions displayed in Supplementary Table 1 guided the analysis.

## Materials and methods

Deidentified data was obtained from “OUT: The National Cancer Survey,” a web-based survey conducted by the National LGBT Cancer Network in the USA between September 2020 and March 2021 [21]. The survey collected information on mental, physical, and social health and the participants’ previous experiences with healthcare providers and institutions. The Western Institutional Review Board (IRB) approved the “OUT: The National Cancer Survey.” Temple University’s IRB provided an exemption for the secondary data analysis.

### Procedures and participants

A non-probability sample of SGM cancer survivors was recruited through community-based methods. The survey was advertised via social media, community networks, and cancer centers. To improve the representation of people of color, the study directors worked closely with community centers that serve SGM individuals from racial and ethnic minority groups, who helped disseminate the survey. Those interested in participating accessed the survey with a link. First, questions to confirm eligibility were presented. Those eligible completed the anonymous 30-min survey.

To be included in the study, participants had to (1) have been diagnosed with cancer, (2) be 18 years or older, (3) self-identify as an LGBTQI + person, and (4) currently live in the USA. Participants who did not speak and read English or Spanish were excluded from the study. For the secondary analysis, we only included participants that reported being diagnosed with anal or colorectal cancer.

Four hundred eleven participants with a previous colorectal or anal cancer diagnosis started the survey. However, only 295 answered the quantitative variable of interest (number of days with poor mental health). We analyzed quantitative differences in clinical, demographic, psychosocial, and patient satisfaction variables among the total sample (*n* = 411) and the sub-sample with the variable of interest (*n* = 295). The total and sub-samples showed consistent data distribution and significance (see Supplementary Tables 2 and 3). Thus, we assumed that data was missing at random and continued the analysis (quantitative and qualitative) with the 295-participant sub-sample.

### Measurement

#### Descriptive data

Demographic information included the participants’ age, sex assigned at birth, gender, sexual orientation, race, ethnicity, employment, area of residence, disability, education, and health insurance. The following clinical data was collected: type of cancer, stage of cancer (i.e., active treatment, remission), having cancer in multiple organs, and receiving more than one cancer diagnosis. Patient satisfaction was assessed using a five-point Likert scale and included questions about the level of satisfaction with the cancer treatment experience (from *very satisfied* to *very dissatisfied*) and receiving culturally competent care by cancer providers, nurses, and healthcare staff (from *all* to *none*).

#### Quantitative data

Mental health was assessed with the question, “Now, think about your mental health, which includes stress, depression, and problems with emotions. In the past 30 days, for how many days was your mental health poor?” In addition, the participants were asked if they had received resources related to mental health for LGBTQI + individuals (*yes*/*no*) and how valuable would be receiving those resources (from *very valuable* to *not very valuable*).

Social health was assessed using Likert-type questions on the participants’ current number of close friends (from *none* to *7 or more*), their perceived level of support before cancer diagnosis (from *very strong* to *very weak*), and changes to the strength of their social network after cancer diagnosis (from *social network being much stronger* to *much weaker*). Additionally, the survey included a question about whether the participants had a primary support person (*yes*/*no*), and if answered “yes,” who this support person was (e.g., current partner, former partner, family member, etc.) and their comfort level bringing the support person to healthcare visits (from *very comfortable* to *very uncomfortable*).

#### Qualitative data

To qualitatively examine the impact cancer had on psychosocial health, the survey included four open-ended questions: (1) How has cancer changed the way you view yourself?; (2) how has cancer changed the way you view yourself as an LGBTQI + person?; (3) how has cancer changed your intimate relationships with others?; and (4) is there anything else you would like to share with us about how cancer has impacted you?

### Data analyses

#### Quantitative data

We used descriptive statistics to assess normality and examine demographic, clinical, patient satisfaction, and psychosocial variables. Group differences were assessed using Mann–Whitney *U* and chi-square tests, depending on the level of measurement. A series of multiple regression models were used to determine contributors to poor mental health days. Based on findings from our previous systematic review of the literature on the psychosocial health of SGM cancer survivors [12], we ran independent models for demographic (model 1), clinical (model 2), and psychosocial (model 3) factors. A fourth model was run with patient satisfaction variables as previous literature on SGM populations with colorectal cancer found these factors influence psychosocial health [16]. Variables that significantly contributed to poor mental health in models 1–4 were included in the final model (model 5). Age and education were forced in the final model since it is already well established that these factors influence the mental health of SGM cancer survivors. Analyses were conducted in RStudio version 2023.09.1 + 494. Alpha was set at 0.05.

#### Qualitative data

For the qualitative data analysis, we followed Brown and Clarke’s [22] process for thematic analysis: (1) two coders (OFR-KT) read all the qualitative responses to become familiar with the data; (2) for each type of cancer (anal or colorectal) and SGM group (SMM, SMW, and TGNB people), the coders independently created inductive, nominal codes in Excel; (3) the emerging codes were grouped into potential themes by each coder independently; (4) the two coders then met to discuss, review, define, and name the themes; (5) finally, all authors revised and agreed on the final themes. In Supplementary Table 4, we acknowledge our positionality and background, which guided a reflection and dialog about our privileges and biases and how they influenced the qualitative data analysis.

#### Data integration

Finally, we integrated the quantitative and qualitative findings at the interpretation and reporting stage [23]. To identify patterns and complement each type of data, we conducted an integration through the narrative, weaving approach, where we present the quantitative and qualitative data together on a concept-by-concept basis [19, 23]. To do this, we first consolidated each data type individually, then merged the findings, and iteratively reworked the concepts until all dimensions were combined following Guetterman et al. [24] recommendations.

## Results

### Descriptive data

The median age of the participants was 60 years. As shown in Table 1, most were males assigned at birth (79.7%) and identified as gay (73.5%) cisgender men (80.3%). The majority reported White/European American race (83.7%), non-Hispanic ethnicity (90.8%), living in urban or suburban areas (83.4%), at least a college or vocational degree (71.5%), having private health insurance (52.9%), and not having a disability (60.7%). Although few participants reported a current diagnosis of cancer (20.7%) or cancer in multiple organs (36.6%), more than half of the sample had received a cancer diagnosis twice or more (54.9%). Importantly, most of the participants indicated being somewhat or very satisfied with their care (89.8%) and receiving culturally competent care from all or most of their cancer care providers (89.8%), nurses (91.2%), and healthcare staff (84.4%). Among demographic, clinical, and patient satisfaction characteristics, only sex, gender, and sexual orientation were significantly different between anal and colorectal SGM cancer survivors (see Table 1).

**Table 1:** Differences in sociodemographic, clinical, psychosocial, and patient satisfaction variables between sexual and gender minority people with anal and colorectal cancer

	Total sample (*n* = 295)	Anal cancer group (*n* = 133)	Colorectal cancer group (*n* = 162)	Statistics		*p*-value
	**Frequency (%)**	**Frequency (%)**	**Frequency (%)**			
*Sociodemographic characteristics*						
Median age in years (IQR)	60.0 (54.0, 80.0)	60.0 (55.0, 65.3)	59.5 (53.8, 65.3)	*U* = 11,080		0.470
Sex				*X*^2^ = 25.042		< 0.001
Female	52 (17.6)	9 (6.8)	43 (26.5)			
Male	235 (79.7)	122 (91.7)	113 (69.8)			
Intersex	5 (1.7)	0 (0.0)	5 (3.1)			
Not reported	3 (1.0)	2 (1.5)	1 (0.6)			
Gender				*X*^2^ = 25.878		< 0.001
Female	46 (15.6)	8 (6.0)	38 (23.5)			
Male	237 (80.3)	123 (92.4)	114 (70.4)			
Transgender	7 (2.4)	1 (0.8)	6 (3.7)			
Genderqueer or non-conforming	3 (1.0)	0 (0.0)	3 (1.9)			
Non-binary	1 (0.3)	1 (0.8)	0 (0.0)			
Another	1 (0.3)	0 (0.0)	1 (0.6)			
Sexual orientation				*X*^2^ = 23.121		< 0.001
Gay	217 (73.5)	114 (85.7)	103 (63.6)			
Lesbian	35 (11.9)	5 (3.8)	30 (18.5)			
Bisexual	16 (5.4)	6 (4.5)	10 (6.2)			
Pansexual	9 (3.1)	1 (0.8)	8 (4.9)			
Queer	18 (6.1)	7 (5.3)	11 (6.8)			
Race				*X*^2^ = 1.571		0.814
White/European American	247 (83.7)	111 (83.5)	136 (84.0)			
Native American	12 (4.1)	4 (3.0)	8 (4.9)			
Black/African American	11 (3.7)	5 (3.8)	6 (3.7)			
Multiracial	10 (3.4)	6 (4.5)	4 (2.5)			
Other	11 (3.7)	5 (3.8)	6 (3.7)			
Not reported	4 (1.4)	2 (1.5)	2 (1.2)			
Ethnicity				*X*^2^ = 3.104		0.078
Hispanic	16 (5.4)	11 (8.3)	5 (3.1)			
Non-Hispanic	268 (90.8)	115 (86.5)	153 (94.4)			
Not reported	11 (3.7)	7 (5.3)	4 (2.5)			
Area of residence				*X*^2^ = 2.560		0.272
Urban	121 (41.0)	48 (36.1)	73 (45.1)			
Suburban	125 (42.4)	62 (46.6)	63 (38.9)			
Rural or remote	46 (15.6)	22 (16.5)	24 (14.8)			
Not reported	3 (1.0)	1 (0.8)	2 (1.3)			
Having a disability (mental, sensory, mobility, cognitive)				*X*^2^ = 2.976		0.084
Yes	116 (39.3)	60 (45.1)	56 (34.6)			
No	179 (60.7)	73 (54.9)	106 (65.4)			
Education				*X*^2^ = 5.517		0.238
Some high school	2 (0.7)	1 (0.8)	1 (0.6)			
High school diploma	14 (4.7)	6 (4.5)	8 (4.9)			
Some college or vocational school	64 (21.7)	34 (25.6)	30 (18.5)			
College/vocational school degree	113 (38.3)	54 (40.6)	59 (36.4)			
Graduate school	98 (33.2)	35 (26.3)	63 (38.9)			
Not reported	4 (1.4)	3 (2.3)	1 (0.6)			
Health insurance				*X*^2^ = 5.192		0.158
Private	156 (52.9)	62 (46.6)	94 (58.0)			
Medicaid	46 (15.6)	22 (16.5)	24 (14.8)			
Medicare	64 (21.7)	36 (27.1)	28 (17.3)			
Other	19 (6.4)	9 (6.9)	10 (6.2)			
Not reported	10 (3.4)	4 (3.0)	6 (3.7)			
*Clinical characteristics*						
Current diagnosis of cancer				*X*^2^ = 0.186		0.665
Yes	61 (20.7)	26 (19.5)	35 (21.6)			
No	218 (73.9)	102 (76.7)	116 (71.6)			
Not reported	16 (5.4)	5 (3.8)	11 (6.8)			
Cancer in multiple organs				*X*^2^ = 1.990		0.158
Yes	108 (36.6)	55 (41.4)	53 (32.7)			
No	187 (63.4)	78 (58.6)	109 (67.3)			
Number of times diagnosed with cancer				*X*^2^ = 0.011		0.913
Only once	133 (45.1)	59 (44.4)	74 (45.7)			
Two or more	162 (54.9)	74 (55.6)	88 (54.3)			
*Patient satisfaction*						
Provision of culturally competent care by cancer care providers				*X*^2^ = 1.478		0.477
All	157 (53.2)	77 (57.9)	80 (49.4)			
Most or some	108 (36.6)	47 (35.3)	62 (38.3)			
Very few or none	21 (7.1)	8 (6.0)	13 (8.0)			
Not reported	8 (2.7)	1 (0.8)	7 (4.3)			
Provision of culturally competent care by nurses				*X*^2^ = 0.592		0.743
All	164 (55.6)	78 (58.6)	86 (53.1)			
Most or some	105 (35.6)	47 (35.3)	58 (35.8)			
Very few or none	18 (6.1)	7 (5.3)	11 (6.8)			
Not reported	8 (2.7)	1 (0.8)	7 (4.3)			
Provision of culturally competent care by healthcare staff				*X*^2^ = 2.727		0.255
All	162 (54.9)	79 (59.4)	83 (51.2)			
Most or some	87 (29.5)	40 (30.1)	47 (29.0)			
Very few or none	23 (7.8)	7 (5.3)	16 (9.9)			
Not reported	23 (7.8)	7 (5.3)	16 (9.9)			
Level of satisfaction with the cancer treatment experience				*X*^2^ = 3.970		0.137
Very/somewhat satisfied	265 (89.8)	118 (88.7)	147 (90.7)			
Neither satisfied nor dissatisfied	9 (3.1)	7 (5.3)	2 (1.2)			
Somewhat/very dissatisfied	19 (6.4)	8 (6.0)	11 (6.8)			
Not reported	2 (0.7)	0 (0.0)	2 (1.2)			
*Mental health*						
Median number of days with poor mental health in the last month (IQR)	5 (1–15)	5 (0–15)	5 (2–15)	*U* = 9639	0.281	
Received mental health resources for LGBTQI + individuals				*X*^2^ = 0.819	0.365	
Yes	94 (31.9)	46 (34.6)	48 (29.6)			
No	193 (65.4)	82 (61.7)	111 (68.5)			
Not reported	8 (2.7)	5 (3.8)	3 (1.9)			
Value of receiving mental health resources for LGBTQI + individuals				*X*^2^ = 6.337	0.096	
Very valuable	136 (46.1)	70 (52.6)	66 (40.7)			
Somewhat valuable	99 (33.6)	39 (29.3)	63 (38.9)			
Not very valuable	44 (14.9)	20 (15.0)	22 (13.6)			
Not reported	15 (5.1)	4 (3.1)	11 (6.8)			
*Social health*						
Current number of friends				*X*^2^ = 5.034	0.283	
None	10 (3.4)	2 (1.5)	8 (4.9)			
1–2 close friends	92 (31.2)	44 (33.1)	48 (29.6)			
3–4 close friends	115 (39.0)	50 (37.6)	65 (40.1)			
5–6 close friends	45 (15.3)	18 (13.5)	27 (16.7)			
7 or more close friends	30 (10.2)	17 (12.8)	13 (8.0)			
Not reported	3 (1.0)	2 (1.5)	1 (0.6)			
Strength of support before cancer diagnosis				*X*^2^ = 9.300	0.009	
Very/somewhat strong	226 (76.6)	112 (84.2)	114 (70.4)			
Neither strong nor weak	49 (16.6)	13 (9.8)	36 (22.2)			
Somewhat/very weak	17 (5.8)	6 (4.5)	11 (6.8)			
Not reported	3 (1.0)	2 (1.5)	1 (0.6)			
Changes in the strength of support after cancer diagnosis				*X*^2^ = 0.803	0.669	
Much/somewhat stronger	127 (43.1)	60 (45.1)	67 (41.4)			
No change	134 (45.4)	59 (44.4)	75 (46.3)			
Somewhat/much weaker	31 (10.5)	12 (9.0)	19 (11.7)			
Not reported	3 (1.0)	2 (1.5)	1 (0.6)			
Having a primary support person				*X*^2^ = 1.550	0.213	
Yes	257 (87.1)	121 (91.0)	136 (84.0)			
No	35 (11.9)	12 (9.0)	23 (14.2)			
Not reported	3 (1.0)	0 (0.0)	3 (1.9)			
Relationship with the support person				*X*^2^ = 3.396	0.493	
Current partner	37 (12.5)	20 (15.0)	17 (10.5)			
Former partner	4 (1.4)	1 (0.8)	3 (1.9)			
Family member	24 (8.1)	14 (10.5)	10 (6.2)			
Friend	30 (10.2)	15 (11.3)	15 (9.3)			
Various (partner and/or family)	161 (54.6)	71 (53.4)	90 (55.9)			
Not reported	39 (13.2)	12 (9.0)	27 (16.7)			
Comfort level bringing the support person to healthcare visits				*X*^2^ = 1.918	0.383	
Comfortable/very comfortable	214 (72.5)	99 (74.4)	115 (71.0)			
Neither comfortable nor uncomfortable	14 (4.7)	5 (3.8)	9 (5.6)			
Uncomfortable/very uncomfortable	8 (2.7)	2 (1.5)	6 (3.7)			
Not applicable/not reported	59 (20.0)	27 (20.3)	32 (19.8)			

The “Not reported” categories were not included in the comparison tests

### Overview of quantitative and qualitative findings before data integration

The median number of days with poor mental health was five. As displayed in Table 1, 65.4% of the participants did not receive mental health resources for LGBTQI + individuals, but almost half of the sample (46.1%) thought this information would have been very valuable. The final model explained 15.8% of the variance (see Table 2). A higher number of days with poor mental health were associated with a current diagnosis of cancer, having a disability, and having fewer close friends.

**Table 2:** Factors associated with number of days with poor mental health among sexual and gender minority populations with anal and colorectal cancer

Variable	Model 0	Model 1, demographic characteristics	Model 2, clinical characteristics	Model 3, psychosocial factors	Model 4, patient satisfaction	Model 5, final contributors
Explained variance by the model (*R*^2^)		*R*^2^ = 0.90*	*R*^2^ = 0.041*	*R*^2^ = 0.038	*R*^2^ = 0.036*	*R*^2^ = 0.158*
Age	− 0.009	0.00 (− 0.01, 0.01)				− 0.01 (− 0.02, 0.00)
Gender						
Cisgender male	0.008	− 0.32 (− 1.08, 0.44)				
Cisgender female	0.002	− 0.42 (− 1.11, 0.27)				
Gender expansive		1.00				
Sexual orientation						
Gay	0.007	− 0.08 (− 0.51, 0.34)				
Lesbian	0.005	0.22 (− 0.34, 0.78)				
Other		1.00				
Racial or ethnic minority						
Yes	0.009	− 0.17 (− 0.49, 0.15)				
No		1.00				
Area of residence						
Urban	0.002	0.22 (− 0.14, 0.59)				
Suburban	0.003	0.13 (− 0.24, 0.49)				
Rural		1.00				
Disability						
Yes	0.062*	0.55 (0.30, 0.79)*				0.42 (0.19, 0.65)*
No		1.00				1.00
Education						
College or higher degree	0.001	− 0.15 (− 0.42, 0.13)				− 0.10 (− 0.35, 0.15)
Less than college		1.00				1.00
Health insurance						
Private		1.00				
Other	0.003	− 0.04 (− 0.29, 0.21)				
Cancer type						
Anal			1.00			
Colorectal	0.001		0.04 (− 0.19, 0.27)			
Current cancer diagnosis						
Yes	0.020*		0.36 (0.08, 0.64)*			0.32 (0.04, 0.60)*
No			1.00			1.00
Cancer in multiple organs						
Yes	0.007		0.35 (0.06, 0.64)*			0.19 (− 0.05, 0.43)
No			1.00			1.00
Number of cancer diagnoses						
Only once			1.00			
Two or more	0.002		− 0.28 (− 0.57, 0.00)			
Received mental health resources for LGBTQI + people						
Yes	0.004			0.08 (− 0.17, 0.32)		
No				1.00		
Number of close friends						
Two or less	0.032*			0.38 (0.07, 0.69)*		0.55 (0.26, 0.84)*
Three or four	0.001			0.19 (− 0.11, 0.48)		0.26 (− 0.02, 0.54)
Five or more				1.00		1.00
Social support before cancer diagnosis						
Strong				1.00		
Nor weak/strong, weak, very weak	0.015*			− 0.14 (− 0.44, 0.16)		
Social support after cancer diagnosis						
Stronger				0.07 (− 0.16, 0.30)		
Nor weaker or stronger, weaker, much weaker	0.000			1.00		
Having a primary support person						
Yes	0.012			− 0.12 (− 0.50, 0.26)		
No				1.00		
Culturally competent care by cancer providers						
All					1.00	1.00
Some, few, none	0.029*				0.39 (0.01, 0.77)*	0.22 (− 0.01, 0.44)
Culturally competent care by nurses						
All					1.00	
Some, few, none	0.015*				− 0.06 (− 0.40, 0.29)	
Culturally competent care by staff						
All					1.00	
Some, few, none	0.022*				0.03 (− 0.34, 0.40)	
Patient satisfaction						
Very/somewhat satisfied					1.00	
Neither satisfied nor dissatisfied, dissatisfied	0.002				0.08 (− 0.37, 0.53)	

Stars (*) indicate significance at the 0.05 level*.* $\beta$loads were standardized. The numbers in parentheses are the 95% confidence intervals. The final model (model 5) was significant (*R*^2^ = 0.1578, *F* [8, 256] = 5.9, *p* < .001) and explained 15.8% of the variance

As displayed in Table 3, two final themes emerged. The first theme, *interactions with others after cancer*, describes how the illness made participants more aware of and influenced their interpersonal relationships. The second theme, *cancer’s influence on physical and mental health*, shows the positive (e.g., increased self-confidence and healthy lifestyles) and negative (e.g., feelings of shame and regret) consequences of cancer and its treatment on the participants’ physical and mental well-being.

**Table 3:** Themes, categories, subcategories, and supporting quotes of the qualitative data analysis

Theme	Category	Subcategory	Supporting quotes
**Interactions with others after cancer**	Romantic and sexual relationships	Physical impacts	“The removal of my prostate has nearly destroyed my sex life. I have a very loving and supportive husband but I still feel humiliated and less than enough for him. This has been the hardest part of both colon cancer and prostate cancer.”
		Psychosocial impacts	Feeling “no longer desirable” and “gross.”
		Relationship status	“Yes radiation made bodily changes that will require surgery in the future but we do other things to stay intimate in the meantime.”
		Ended relationships	“My partner broke up with me after 22 years. Some of the statements, you can’t support yourself, I don’t want to be responsible for your debt, and I’m sick of you being sick.”
	Interactions with the LGBTQI + community	Decreased engagement with the community	“There is a stigma of being a cancer survivor, specifically a colorectal cancer survivor that sex will be a problem. There is a stigma in general with a cancer survivor somewhat like being an HIV positive man of years ago. It causes men to question if you are someone they want to date based on that alone.”
		Out and proud	“The community needs to come together as a unit like other communities and you can’t exclude any of the L G B T Q and beyond members. I felt there was nowhere to turn. Luckily, my attitude and ability to move beyond has been there but after cancer I began to worry for my other brothers and sisters.” “[I am] more passionate about what I leave for the generations after. What I can do [for them].”
	Non-intimate relationships	Strengthened	“Cancer changed my intimate relationship with others in that folks know they can come to me and talk about anything. Best friends became family. Acquaintances became friends. Strangers became part of my support system.” “I am able to see what is truly important in life, which is our relationships and our family and happiness. I avoid investing too much time in people who don’t share those priorities.”
		Weakened	“I grew up in a tight knit extended family of 35 1st cousins. I know them all personally and after my cancer, I only have 2 that call me and only 3 that will take my calls. No one in the extended family called me during treatment and I’m not allowed to talk about it now.” “I am more sensitive, more withdrawn, more inward.”
	Interactions with healthcare providers	–	“Yes, it was very important for me to have oncologists that realized that my anus serves as a sexual organ.” “It’s ruined me. Of the many things I was not aware of, is how barbaric medicine really still is. Hacking and cutting with no control over what other organs they damage. Having it not even occur to them or care whether they’re cutting someone’s pleasure center out and throwing it in a bucket. All of it was a relatively ugly experience including robotic nurses who could not have given their last fuck whether I lived or died, and pompous condescending doctors who view you as a slab of flesh to be cut, “fixed,” and processed out…”
**Cancer’s influence on physical and mental health**	Change of perspective	Mortality	“I’m far less concerned about my ultimate passing. It shouldn’t be from the cancer I had, but hopefully from old age. I’m more at peace with myself, I think. I dodged a bullet.”
		Gratitude	“I’m much more empathetic, and I do feel this extreme gratitude to be alive.”
		Priorities	“Before cancer, I always had the attitude of Carpe Diem and the cancer experience intensified that. Life is a precious gift!!!”
		LGBTQI + identity	“Now that I feel stronger, I feel that I am able to withstand any stigma I might face and do what I need to be happy.”
		Self-confidence	“I am ecstatic to be back at college and learning and have a greater passion for life!! I always did and so important to live in the moment with plans, to tell the people you love, I love and appreciate you, and to find the good on people through empathy. The biggest lesson for me was I too deserve the above and am learning to give that to myself!”
	Negative effects on mental health	Self-esteem and self-image	“I do not like the way I look. I was in body building. When I became sick I lost 80lbs. I do not like what I see in the mirror.”
		Psychological distress	“I am angry with myself for not taking care of getting rid of my anal warts which led to my cancer.”
		Fear of recurrence	“I’m much more careful about possible reinfection of HPV, which can be a cause of cancer.” “It has impacted me to the extent that I know that it could (and probably will) come back at some point at a time when I’m less healthy and able to handle the treatment process. This is, I think, a reasonably worry.”
	Minority stress	Internalized	“The fact that it’s ANAL cancer shames me. So I stay isolated about it. Not worrying family.”
		Experiences	“We, as a group, are always sexualized as opposed to being seen as a multi faceted person and being gay is only one facet. I will never understand this and I don’t want to know what they do in the privacy of their homes but they feel entitled to pretend to know what happens in mine and judge me for that.”
	Physical factors	–	“Cancer surgery was a nightmare.. Being HIV + my immune response wasn’t great for healing and it took nearly a year to heal from surgery.” “Because I am also HIV + I am more keenly aware of the very negative impact that radiation has had on my immune system… low CD4 counts during a pandemic are nerve-wracking.”
	Response and adaptations to cancer	Positive mindset	“Humor,” “pushing through,” “remaining positive,” and an overall “fuck you cancer!” attitude
		Advocacy	“I have been very active in helping others through this journey. I have spoken at a nursing oncology conference concerning the gay cancer patient. I feel that it is now my calling. I was stage 3 and very lucky to be alive.” “More passionate about what I leave for the generations after. What I can do [for them].”
	Lasting impact	–	“No one ever explained how my body would change and what I would have to deal with on my own. Many of the side [effects] have been difficult to deal with and I don’t know where to get help.” “I don’t feel healthy and strong anymore because of the side effects of treatment that will probably only get worse with time.” “I got a brain injury from the FOLFOX […]. It took me 6 years to rebuild my brain with the help of amazing team mostly SLPs. Cancer stole so much from me!”

### Data integration

Three concepts emerged from the data integration. First, there were positive and negative psychological effects attributed to cancer as described by the participants (e.g., feeling “stronger” and “pushing through” but also “angry” and “not liking what [they] see in the mirror”). Second, cancer required the participants to make social adaptations with their friends, partners, and family members, such as prioritizing fewer and strong connections over multiple but poor-quality relationships. Finally, several factors affected the psychosocial health of the participants during and after cancer, particularly interactions with providers (e.g., poor communication about the long-term effects of cancer treatment), comorbidities, and lack of knowledge to manage treatment side effects.

#### Psychological effects of cancer

Only 31.9% of the participants received mental health resources tailored for LGBTQI + individuals, which may explain some of the detrimental effects on SGM survivors’ self-esteem. Many of these negative effects were associated with body image due to cancer treatment. SGM participants reported feeling “broken,” “unlovable,” “destroyed,” and “no longer… a whole.” In addition, cancer generated symptoms of “anxiety,” “depression,” “fear,” “anger,” “trauma,” and among SMM, fear of HPV reinfection and cancer recurrence.

Interestingly, 79.7% of the participants considered tailored LGBTQI + mental health resources valuable. Still, since most did not receive it, they developed their own coping mechanisms to make sense of their diagnosis and support other LGBTQI + cancer survivors. Specifically, they expressed having a positive mindset, such as “humor,” “pushing through,” “remaining positive,” and an overall “fuck you cancer!” attitude. Participants became more acutely aware of the unique needs of LGBTQI + individuals, and for SMM and SMW, this sparked a “calling” to advocate for others, which manifested as promoting screening, mentoring younger generations, and being a spokesperson for LGBTQI + cancer survivors. Other participants, particularly SMW and TGNB individuals, reported disengagement with the community because they did not feel supported, and instead, felt ostracized due to the impact of cancer treatment on their health and well-being.

A higher number of days with poor mental health were associated with a current diagnosis of cancer ($\beta$ = 0.32, 95% CI = 0.04–0.60). SGM survivors expressed that cancer stimulated an inner dialog about mortality. In particular, feelings of limited time and looming death fostered an awareness of mortality, which was more pronounced during active cancer. Consequently, cancer made those in remission see “every day as a gift” and feel that they had a “second opportunity” and overall appreciation and gratitude for being alive. The appreciation for life also encouraged SGM individuals to “stay healthy” and be more active in caring for their physical and mental health. Physically, participants mentioned improving their diet, exercising more, and taking proactive screening measures. Mentally, the participants felt “stronger” and a sense of pride for being a “survivor.” They were also more confident, showed more love and compassion for themselves, and cared less about what others thought.

#### Social adaptations

The strength of support before cancer diagnosis was strong for 76.6% of the sample and did not change (45.4%) or was stronger (43.1%) after cancer diagnosis for most participants, consistent with the qualitative data, which shows that cancer deepened and strengthened the interpersonal relationships of the participants across all SGM groups. Sometimes, this was at the cost of casual or negative relationships. They had fewer but stronger relationships, also consistent with the quantitative findings, where only 25.5% of the participants reported five or more close friends after cancer diagnosis. Despite this, a few SGM survivors had a weak support network after cancer (10.5%), which may be due to negative experiences with friends and family. In particular, SMM and TGNB participants felt withdrawn, lonely, and isolated—feelings exacerbated during the COVID-19 pandemic. Having fewer close friends was associated with more mentally unhealthy days ($\beta$ = 0.55, 95% CI = 0.26–0.84).

Importantly, 67.1% of the participants mentioned their partner was their support person during their cancer journey, and even though SMM and SMW felt closer to their partners after diagnosis, some felt guilty for the burden cancer had placed on their partner. Importantly, TGNB participants were the only group who expressed that cancer ended their romantic relationships. All in all, cancer and its treatment had detrimental effects on the intimacy of the participants regardless of SOGI and cancer type. Physically, sex was perceived as “painful,” “undesirable,” or challenging compared to before cancer. Psychologically, cancer negatively affected their self-esteem and made them feel “no longer desirable” and “gross.” Although negative self-perceptions affected both single individuals and those in long-term relationships, those who were single seemed to have much lower self-esteem and a general lack of hope. Many of these individuals took an avoidant approach and stated they could or would not have sex or intimate relationships ever again. SMM individuals, notably those diagnosed with anal cancer, stated that their fear of infecting future partners with HPV and potentially “caus[ing] anyone else cancer” was a reason for their disengagement in romantic relationships. Furthermore, SMM at older ages used their age to justify loss of intimacy.

#### Factors affecting psychosocial health during and after cancer

Many SGM survivors experienced stigma related to their cancer diagnosis and queer identity. For some, there was already a lot of shame and stigma before diagnosis, and cancer did not change that. Internalized stigma made the cancer journey more difficult, and experiences with providers exacerbated these feelings. For instance, SMM cancer survivors were disappointed by their healthcare providers’ lack of knowledge regarding LGBTQI + health and infection-related cancers. They also wished providers would have been more upfront about the impact of cancer treatment on their sexual functioning. TGNB participants expressed that their identities were “judged,” reduced to their sexuality, and wished they were seen as “multi-faceted” and holistic beings. Despite this, most participants referred that at least half of their cancer providers, nurses, and healthcare staff provided culturally competent care. Similarly, 89.9% felt satisfied with their care, possibly due to some positive experiences with providers, particularly communication about their needs and treatment expectations.

The participants also reported other health conditions and comorbidities that impacted their cancer journey to different extents. For some, their comorbidities (e.g., cardiopulmonary problems) were their main concern, and cancer was a “minor inconvenience.” In contrast, others felt cancer was “tougher” than any other health condition they had ever experienced. Similarly, some participants living with HIV felt cancer had affected them to a lower degree because they had already developed skills to face life-threatening conditions. However, participants with concurrent cancer and HIV diagnoses were worried about the impact of cancer treatment (e.g., surgery and radiotherapy) on their immune systems.

Although most of the participants were middle-aged or young-old adults, the participants reported feeling “older,” “weaker,” “tired,” and “physically and behaviorally damaged.” Furthermore, SGM cancer survivors described the long-term impact of cancer and associated treatments on their general health. They felt unprepared and uneducated about the likelihood of developing these side effects and how to handle them. For instance, participants experienced symptoms such as neuropathy, pain, diarrhea, memory and sleep problems, and sexual side effects even after finishing treatment, which may be why the quantitative results indicated that having a disability (e.g., sensory, mobility, mental, cognitive, or developmental) was also associated with more poor mental health days ($\beta$ = 0.42, 95% CI = 0.19–0.65).

## Discussion

GI cancers and their treatment have a profound effect on the psychosocial health of SGM individuals. Both anal and colorectal cancer survivors reported a median of 5.0 days with poor mental health in the last month, which is 0.9 and 0.7 days more than the average for the US population in 2020 and 2021, respectively [25]. Participants expressed feelings of depression, anxiety, anger, and trauma due to changes in their bodies, which is consistent with the existing literature [13, 14, 16]. Notably, participants were interested in improving their mental health. For instance, almost 80% of the participants would have valued receiving tailored LGBTQI + mental health resources. However, less than a third received them. Despite this, the majority of those sampled expressed satisfaction with their care and perceived most of their providers as culturally sensitive. But again, through qualitative inquiry, some participants expressed that healthcare providers poorly communicated the side effects of cancer treatment and lacked training on the healthcare needs of LGBTQI + individuals. All in all, our findings evidence a mismatch between perceived and actual provision of cultural care.

The trauma-informed care perspective proposes that SGM individuals experience trauma throughout the cancer care continuum [26]. In this analysis, SGM cancer survivors mentioned that previous experiences of discrimination increased their vulnerability to poor psychosocial health during the cancer trajectory. Specifically, we found that traumatic experiences—lived experiences and historical trauma (e.g., stigma against SGM groups during the HIV epidemic)—before diagnosis made participants anticipate and experience poor healthcare quality during their cancer journey. Educational programs for healthcare providers and staff should consider historical and ongoing stigma when developing, implementing, and assessing culturally sensitive training programs; this will allow the provision of high-quality, trauma-informed care.

Previous research has found that SMW breast cancer survivors are more likely to engage in active coping strategies than their heterosexual cisgender counterparts [27–29]. Although our study did not include a heterosexual cisgender comparison group and most of our participants were SMM, they also expressed a positive mindset (e.g., humor), improved their lifestyles, and “pushed through” cancer, indicating that other SGM groups also engage in active coping strategies, even despite the lack of targeted mental health resources. Intervention studies aimed at improving the psychosocial health of SGM anal and colorectal cancer survivors should consider this protective factor when treating the late and long-term effects of cancer.

The participants also mentioned being more outspoken about the needs of members of their community and, in many cases, advocated for cancer prevention and screening among their friends, suggesting that peer support interventions may improve the psychosocial health of SGM cancer survivors. To the best of our knowledge, studies analyzing peer-to-peer support programs among this population are lacking, even though previous research has shown that peer support interventions are beneficial for the mental health and quality of life of cancer survivors [30] and SGM populations [31].

Differences were noted between SGM subgroups based on cancer type. Consistent with studies suggesting that the incidence of anal cancer is higher among SMM [32], our findings show that anal cancer survivors were more likely to be males assigned at birth and identify as gay cisgender men, compared to SMW and TGNB participants. Anal cancer survivors also had a weaker social network than colorectal survivors before diagnosis and mentioned feelings of shame and regret due to their diagnosis. Professional organizations and advocacy groups have proposed that the stigma anal cancer survivors face may be due to intersectional stigmas associated with the anus, sexual practices involving receptive anal intercourse, and risk factors for anal cancer, particularly HPV infection and multiple sexual partners [33–35]. All in all, our findings indicate that SGM anal cancer survivors may be at a higher risk of poorer psychosocial health. Future research is necessary to expand on the impact of these subgroups and propose interventions to address their psychosocial needs.

SGM survivors had mixed feelings about the impact of cancer on their romantic relationships. Some felt that their cancer diagnosis made them closer to their partner, but others felt it placed a burden on them. Furthermore, the impact of cancer on the sexual functioning of the participants created additional barriers to intimacy, which is similar to previous research findings [36, 37]. Nonetheless, we also identified that single SGM survivors lacked the confidence to pursue romantic relationships; they seemed to have lower self-esteem and a general lack of hope. In this sense, and consistent with the general cancer literature, having a partner may protect against worse cancer outcomes [38]. Interventions for improving self-esteem and helping those interested in pursuing romantic relationships are necessary to improve the overall psychosocial health of SGM people with cancer.

Through qualitative inquiry, sexual minorities were the only group that expressed the impact of cancer on their physical health and queer identity. SMM also shared their fear of cancer recurrence and internalized minority stress. Finally, TGNB participants described the detrimental impact cancer had on their family and romantic relationships, most of which ended after the diagnosis. These findings are similar to those of previous studies [8, 39, 40] but also expand on the unique psychosocial needs of SGM GI cancer survivors. In particular, participants from our study expressed feelings of internalized stigma due to being diagnosed with an infectious-related cancer and the detrimental effects of cancer on their romantic relationships. It is worth noting that although these subcategories emerged for those subgroups only, other SGM subgroups may have similar experiences. Nevertheless, these SGM subgroups may perceive these issues as a priority for their psychosocial well-being; as such, considering the unique needs of SGM subgroups is essential to improve patient-centered care and precision health.

Our participants experienced neuropsychological symptoms broadly studied in the cancer literature but poorly understood among SGM cancer survivors, such as pain, sleeping problems, and cognitive dysfunction. These health domains are essential for daily life activities (e.g., cooking and taking medications) [41], and their disruption can potentially decrease cancer survivorship, job reintegration, and overall quality of life [42–44]. Furthermore, these long-term symptoms are associated with cognitive and mental disabilities, a significant predictor of poor mental health. Research studies analyzing the impact of cancer and its treatment on the neuropsychological health of SGM cancer survivors are urgently needed.

### Study limitations

The external validity of the findings is limited by the non-probability sample. Furthermore, construct validity was limited by the use of open-ended questions which did not allow further probing on emerging themes and the use of single item measures for many variables. Due to the limitations in the length of the survey, many clinically important measures were not included in the dataset. Despite this, we conducted a rigorous qualitative and quantitative analysis, followed by a widely used data integration technique, improving the overall quality of our study. We compared findings among SGM subgroups with different types of cancer, which is rare in the existing literature. Finally, we included a significant sample of GI cancer survivors, particularly anal survivors, which has been previously reported as a hard group to recruit for research studies.

### Conclusion

GI cancers affect the psychosocial health of SGM individuals. Older age, type of cancer, being unpartnered, having a disability, and fewer close friends may increase the risk of poorer psychosocial health. To cope with their diagnosis, SGM survivors developed unique strategies and advocated for the SGM community. Developing and implementing psychosocial interventions aimed at restoring the self-esteem and social support is a priority for improving the psychosocial well-being of SGM GI cancer survivors. These interventions should consider peer-to-peer mechanisms, the intersecting stigmas affecting anal cancer survivors, and the social healthcare needs of TGNB individuals. Finally, training for healthcare professionals should include education on historical and ongoing trauma, and measures and mechanisms should be developed to assess both the perceived and actual provision of culturally responsive care.

## Supplementary Information

Below is the link to the electronic supplementary material.

Supplementary file1 (DOCX 48 KB)

## Data Availability

The datasets generated during and/or analyzed during the current study are available from the corresponding author on reasonable request.
