# Supplementary material for: The psychosocial health of sexual and gender minority people with anal and colorectal cancer: a mixed methods study
Source: J Cancer Surviv. 2024 May 8;19(6):2012–26. doi: 10.1007/s11764-024-01611-5 (PMC12546364; doi:10.1007/s11764-024-01611-5)
Supplement: Supplementary file 1 — Supplementary file1 (DOCX 48 KB) [file 11764_2024_1611_MOESM1_ESM.docx]

**Supplementary Table 1. Research questions and hypotheses.**

| Overarching question: How does gastrointestinal cancer influence the psychosocial health of SGM people with cancer? | |
| --- | --- |
| Type of data or analysis | **Question** |
| Quantitative data | What factors (sociodemographic, clinical, and patient satisfaction) predict the psychosocial health of SGM individuals with GI cancer? |
|  | What factors differ between anal and colorectal SGM cancer survivors? |
| Qualitative data | How do anal and colorectal cancers influence the psychosocial health of SGM people? |
|  | What are the differences in the perceived psychosocial health of anal and colorectal SGM cancer survivors? |
| Quantitative and qualitative data integration | To what extent quantitative and qualitative data converge with each other? |

**Note.** GI, Gastrointestinal; SGM, Sexual and gender minority populations.

**Supplementary Table 2. Differences in sociodemographic and clinical characteristics between sexual and gender minority people with anal and colorectal cancer before excluding cases that did not answer the outcome variable.**

|  | Anal cancer group  (n= 186) | Colorectal cancer group  (n= 225) | Statistics | p | Anal cancer group  (n= 133) | Colorectal cancer group  (n= 162) | Statistics | p |
| --- | --- | --- | --- | --- | --- | --- | --- | --- |
|  | **Frequency (%)** | **Frequency (%)** |  |  | **Frequency (%)** | **Frequency (%)** |  |  |
| *Sociodemographic characteristics* | | | | | | | | |
| Mean age in years (IQR) | 60.0 (54.8 – 65.0) | 59.0 (53.0 – 65.0) | U= 14,866 | 0.365 | 60.0 (55.0 – 65.3) | 59.5 (53.8 – 65.3) | U= 11,080 | 0.470 |
| Sex  Female  Male  Intersex  Not reported | 11 (5.9)  174 (93.5)  0 (0.0)  1 (0.5) | 57 (25.3)  160 (71.1)  8 (3.6)  0 (0.0) | X^2^= 36.146 | <.001 | 9 (6.8)  122 (91.7)  0 (0.0)  2 (1.5) | 43 (26.5)  113 (69.8)  5 (3.1)  1 (0.6) | X^2^= 25.042 | <.001 |
| Gender  Female  Male  Transgender  Genderqueer or non-conforming  Non-Binary  Another | 9 (4.8)  174 (93.5)  1 (0.5)  0 (0.0)  2 (1.1)  0 (0.0) | 51 (22.7)  162 (72.0)  8 (3.6)  3 (1.3)  0 (0.0)  1 (0.4) | X^2^= 37.914 | <.001 | 8 (6.0)  123 (92.4)  1 (0.8)  0 (0.0)  1 (0.8)  0 (0.0) | 38 (23.5)  114 (70.4)  6 (3.7)  3 (1.9)  0 (0.0)  1 (0.6) | X^2^= 25.878 | <.001 |
| Sexual orientation  Gay  Lesbian  Bisexual  Pansexual  Queer | 162 (87.1)  6 (3.2)  8 (4.3)  1 (0.5)  9 (4.8) | 146 (64.9)  43 (19.1)  15 (6.7)  8 (3.6)  13 (5.8) | X^2^= 33.675 | <.001 | 114 (85.7)  5 (3.8)  6 (4.5)  1 (0.8)  7 (5.3) | 103 (63.6)  30 (18.5)  10 (6.2)  8 (4.9)  11 (6.8) | X^2^= 23.121 | <.001 |
| Race  White/European American  Native American  Black/African American  Multiracial  Other  Not reported | 137 (73.7)  5 (2.7)  6 (3.2)  10 (5.4)  7 (3.8)  21 (11.3) | 170 (75.6)  8 (3.6)  11 (4.9)  8 (3.6)  9 (4.0)  19 (8.4) | X^2^= 1.672 | 0.796 | 111 (83.5)  4 (3.0)  5 (3.8)  6 (4.5)  5 (3.8)  2 (1.5) | 136 (84.0)  8 (4.9)  6 (3.7)  4 (2.5)  6 (3.7)  2 (1.2) | X^2^= 1.571 | 0.814 |
| Ethnicity  Hispanic  Non-Hispanic  Not reported | 17 (9.1)  142 (76.3)  27 (14.5) | 9 (4.0)  193 (85.8)  23 (10.2) | X^2^= 4.286 | 0.038 | 11 (8.3)  115 (86.5)  7 (5.3) | 5 (3.1)  153 (94.4)  4 (2.5) | X^2^= 3.104 | 0.078 |
| Area of residence  Urban  Suburban  Rural or remote  Not reported | 58 (31.2)  70 (37.6)  24 (12.9)  34 (18.3) | 81 (36.0)  74 (32.9)  30 (13.3)  40 (17.8) | X^2^= 1.365 | 0.505 | 48 (36.1)  62 (46.6)  22 (16.5)  1 (0.8) | 73 (45.1)  63 (38.9)  24 (14.8)  2 (1.3) | X^2^= 2.560 | 0.272 |
| Having a disability (mental, sensory, mobility, cognitive)  Yes  No | 67 (36.0)  119 (64.0) | 66 (29.3)  159 (70.7) | X^2^= 1.786 | 0.183 | 60 (45.1)  73 (54.9) | 56 (34.6)  106 (65.4) | X^2^= 2.976 | 0.084 |
| Education  Some high school  High school diploma  Some college/vocational school  College/vocational school degree  Graduate school  Not reported | 1 (0.5)  7 (3.8)  39 (21.0)  61 (32.8)  41 (22.0)  37 (19.9) | 3 (1.3)  13 (5.8)  35 (15.6)  65 (28.9)  70 (31.1)  39 (17.3) | X^2^= 6.715 | 0.151 | 1 (0.8)  6 (4.5)  34 (25.6)  54 (40.6)  35 (26.3)  3 (2.3) | 1 (0.6)  8 (4.9)  30 (18.5)  59 (36.4)  63 (38.9)  1 (0.6) | X^2^= 5.517 | 0.238 |
| Health Insurance  Private  Medicaid  Medicare  Other  Not reported | 76 (40.9)  23 (12.4)  38 (20.4)  10 (5.4)  39 (21.0) | 107 (47.6)  32 (14.2)  31 (13.8)  10 (4.4)  45 (20.0) | X^2^= 4.146 | 0.246 | 62 (46.6)  22 (16.5)  36 (27.1)  9 (6.9)  4 (3.0) | 94 (58.0)  24 (14.8)  28 (17.3)  10 (6.2)  6 (3.7) | X^2^= 5.192 | 0.158 |
| *Clinical characteristics* | | | | | | | | |
| Current diagnosis of cancer  Yes  No  Not reported | 33 (17.7)  143 (76.9)  10 (5.4) | 49 (21.8)  162 (72.0)  14 (6.2) | X^2^= 0.897 | 0.343 | 26 (19.5)  102 (76.7)  5 (3.8) | 35 (21.6)  116 (71.6)  11 (6.8) | X^2^= 0.186 | 0.665 |
| Cancer in multiple organs  Yes  No | 82 (44.1)  104 (55.9) | 70 (31.1)  155 (68.9) | X^2^= 6.809 | 0.009 | 55 (41.4)  78 (58.6) | 53 (32.7)  109 (67.3) | X^2^= 1.990 | 0.158 |
| Number of times diagnosed with cancer  Only once  Two or more | 101 (54.3)  85 (45.7) | 128 (56.9)  97 (43.1) | X^2^= 0.181 | 0.670 | 59 (44.4)  74 (55.6) | 74 (45.7)  88 (54.3) | X^2^= 0.011 | 0.913 |
| *Patient satisfaction* | | | | | | | | |
| Provision of culturally competent care by cancer care providers  All  Most or some  Very few or none  Not reported | 102 (54.8)  64 (34.4)  10 (5.4)  10 (5.4) | 111 (49.3)  77 (34.2)  17 (7.6)  20 (8.9) | X^2^= 1.193 | 0.550 | 77 (57.9)  47 (35.3)  8 (6.0)  1 (0.8) | 80 (49.4)  62 (38.3)  13 (8.0)  7 (4.3) | X^2^= 1.478 | 0.477 |
| Provision of culturally competent care by nurses  All  Most or some  Very few or none  Not reported | 103 (55.4)  65 (34.9)  8 (4.3)  10 (5.4) | 116 (51.6)  73 (32.4)  16 (7.1)  20 (8.9) | X^2^= 1.704 | 0.426 | 78 (58.6)  47 (35.3)  7 (5.3)  1 (0.8) | 86 (53.1)  58 (35.8)  11 (6.8)  7 (4.3) | X^2^= 0.592 | 0.743 |
| Provision of culturally competent care by health care staff  All  Most or some  Very few or none  Not reported | 103 (55.4)  56 (30.1)  10 (5.4)  17 (9.1) | 111 (49.3)  66 (29.3)  19 (8.4)  29 (12.9) | X^2^= 1.925 | 0.381 | 79 (59.4)  40 (30.1)  7 (5.3)  7 (5.3) | 83 (51.2)  47 (29.0)  16 (9.9)  16 (9.9) | X^2^= 2.727 | 0.255 |
| Level of satisfaction with the cancer treatment experience  Very/Somewhat satisfied  Neither satisfied or dissatisfied  Somewhat/Very dissatisfied  Not reported | 157 (84.4)  10 (5.4)  11 (5.9)  8 (4.3) | 195 (86.7)  3 (1.3)  13 (5.8)  14 (6.2) | X^2^= 5.276 | 0.071 | 118 (88.7)  7 (5.3)  8 (6.0)  0 (0.0) | 147 (90.7)  2 (1.2)  11 (6.8)  2 (1.2) | X^2^= 3.970 | 0.137 |

**Note.** The “Not reported” categories were not included in the comparison tests.

**Supplementary Table 3. Differences in psychosocial outcomes between sexual and gender minority people with anal and colorectal cancer before excluding cases that did not answer the outcome variable.**

|  | Full sample | | | | Sample without missing cases | | | |
| --- | --- | --- | --- | --- | --- | --- | --- | --- |
|  | **Anal cancer group**  **(n= 186)** | **Colorectal cancer group**  **(n= 225)** | **Statistics** | **p** | **Anal cancer group**  **(n= 133)** | **Colorectal cancer group**  **(n= 162)** | **Statistics** | **p** |
|  | **Frequency (%)** | **Frequency (%)** |  |  | **Frequency (%)** | **Frequency (%)** |  |  |
| *Mental health* | | | | | | | | |
| Received mental health resources for LGBTQI+ individuals  Yes  No  Not reported | 50 (26.9)  98 (52.7)  38 (20.4) | 57 (25.3)  125 (55.6)  43 (19.1) | X^2^= 0.127 | 0.720 | 46 (34.6)  82 (61.7)  5 (3.8) | 48 (29.6)  111 (68.5)  3 (1.9) | X^2^= 0.819 | 0.365 |
| Value of receiving mental health resources for LGBTQI+ individuals  Very valuable  Somewhat valuable  Not very valuable  Not reported | 75 (40.3)  48 (25.8)  21 (11.3)  42 (22.6) | 78 (34.7)  68 (30.2)  28 (12.4)  51 (22.7) | X^2^= 1.692 | 0.638 | 70 (52.6)  39 (29.3)  20 (15.0)  4 (3.1) | 66 (40.7)  63 (38.9)  22 (13.6)  11 (6.8) | X^2^= 6.337 | 0.096 |
| *Social health* | | | | | | | | |
| Current number of friends  None  1-2 close friends  3-4 close friends  5-6 close friends  7 or more close friends  Not reported | 3 (1.6)  57 (30.6)  68 (36.6)  24 (12.9)  20 (10.8)  14 (7.5) | 9 (4.0)  61 (27.1)  84 (37.3)  33 (14.7)  21 (9.3)  17 (7.6) | X^2^= 2.880 | 0.578 | 2 (1.5)  44 (33.1)  50 (37.6)  18 (13.5)  17 (12.8)  2 (1.5) | 8 (4.9)  48 (29.6)  65 (40.1)  27 (16.7)  13 (8.0)  1 (0.6) | X^2^= 5.034 | 0.283 |
| Strength of support before cancer diagnosis  Very/Somewhat strong  Neither strong nor weak  Somewhat/Very weak  Not reported | 143 (76.9)  18 (9.7)  12 (6.5)  13 (7.0) | 150 (66.7)  44 (19.6)  11 (4.9)  20 (8.9) | X^2^= 8.465 | 0.014 | 112 (84.2)  13 (9.8)  6 (4.5)  2 (1.5) | 114 (70.4)  36 (22.2)  11 (6.8)  1 (0.6) | X^2^= 9.300 | 0.009 |
| Changes in the social support after cancer diagnosis  Much/Somewhat stronger  No change  Somewhat/much weaker  Not reported | 72 (38.7)  84 (45.2)  17 (9.1)  13 (7.0) | 90 (40.0)  93 (41.3)  22 (9.8)  20 (8.9) | X^2^= 0.392 | 0.821 | 60 (45.1)  59 (44.4)  12 (9.0)  2 (1.5) | 67 (41.4)  75 (46.3)  19 (11.7)  1 (0.6) | X^2^= 0.803 | 0.669 |
| Having a primary support person  Yes  No  Not reported | 157 (84.4)  17 (9.1)  12 (6.5) | 175 (77.8)  29 (12.9)  21 (9.3) | X^2^= 1.345 | 0.246 | 121 (91.0)  12 (9.0)  0 (0.0) | 136 (84.0)  23 (14.2)  3 (1.9) | X^2^= 1.550 | 0.213 |
| Relationship with the support person  Current partner  Former partner  Family member  Friend  Various (partner and/or family)  Not reported | 25 (13.4)  1 (0.5)  17 (9.1)  17 (9.1)  96 (51.6)  30 (16.1) | 25 (11.1)  3 (1.3)  12 (5.3)  19 (8.4)  114 (50.7)  52 (23.1) | X^2^= 2.644 | 0.618 | 20 (15.0)  1 (0.8)  14 (10.5)  15 (11.3)  71 (53.4)  12 (9.0) | 17 (10.5)  3 (1.9)  10 (6.2)  15 (9.3)  90 (55.9)  27 (16.7) | X^2^= 3.396 | 0.493 |
| Comfort level bringing the support person to healthcare visits  Comfortable/Very comfortable  Neither comfortable or uncomfortable  Uncomfortable/Very uncomfortable  Not applicable/Not reported | 128 (68.8)  7 (3.8)  4 (2.2)  47 (25.3) | 148 (65.8)  9 (4.0)  8 (3.6)  60 (26.7) | X^2^= 0.814 | 0.665 | 99 (74.4)  5 (3.8)  2 (1.5)  27 (20.3) | 115 (71.0)  9 (5.6)  6 (3.7)  32 (19.8) | X^2^= 1.918 | 0.383 |

**Note.** The “Not reported” categories were not included in the comparison tests.

**Supplementary Table 4. Authors positionality**

| Author | Positionality and background |
| --- | --- |
| Oscar Y. Franco-Rocha (He/Él) | Cisgender gay man from a rural town in Colombia, South America. Latino ethnicity, Mestizo race, and Campesino ancestry. First-generation student for both undergraduate and graduate schools.  Doctoral student at the University of Texas at Austin. Research assistant of the Brain Health Neuroscience Lab. His research focuses on the influence of minority stress on the mental health of cancer survivors from the LGBTQ+ community. |
| Katie Trainum (She/Her) | White cisgender heterosexual woman. She was born and raised in the United States.  Doctoral student at the University of Texas at Austin. Her research focuses on healthcare provider job satisfaction and burnout. |
| Christopher W. Wheldon (He/Him) | White cisgender queer man raised by a single parent and first-generation college graduate. He was born and raised in the United States. His research focuses on improving cancer prevention and cancer care delivery for underserved and vulnerable populations. |

Information retrieved from [10] and updated for the present study.
